# Using sequence data to identify alternative routes and risk of infection: a case-study of campylobacter in Scotland

**DOI:** 10.1186/1471-2334-12-80

**Published:** 2012-04-01

**Authors:** Paul R Bessell, Ovidiu Rotariu, Giles T Innocent, Alison Smith-Palmer, Norval JC Strachan, Ken J Forbes, John M Cowden, Stuart WJ Reid, Louise Matthews

**Affiliations:** 1 Institute of Biodiversity, Animal Health and Comparative Medicine, College of Medical, Veterinary and Life Sciences, University of Glasgow, 464 Bearsden Rd, Glasgow, UK; 2 Institute of Biological and Environmental Sciences, School of Biological Sciences, University of Aberdeen, Aberdeen, UK; 3 Health Protection Scotland, National Services Scotland, 4th Floor Meridian Court, 5 Cadogan Street, Glasgow, UK; 4 Section of Immunology and Infection, Polwarth Building, University of Aberdeen, Aberdeen, UK; 5 Royal Veterinary College, University of London, Hawkshead Lane, North Mymms, Hatfield, Hertfordshire, UK; 6 The Roslin institute, The University of Edinburgh, Easer Bush, Edinburgh EH25 9RG, UK

## Abstract

**Background:**

Genetic typing data are a potentially powerful resource for determining how infection is acquired. In this paper MLST typing was used to distinguish the routes and risks of infection of humans with *Campylobacter jejuni *from poultry and ruminant sources

**Methods:**

*C. jejuni *samples from animal and environmental sources and from reported human cases confirmed between June 2005 and September 2006 were typed using MLST. The STRUCTURE software was used to assign the specific sequence types of the sporadic human cases to a particular source. We then used mixed case-case logistic regression analysis to compare the risk factors for being infected with *C. jejuni *from different sources.

**Results:**

A total of 1,599 (46.3%) cases were assigned to poultry, 1,070 (31.0%) to ruminant and 67 (1.9%) to wild bird sources; the remaining 715 (20.7%) did not have a source that could be assigned with a probability of greater than 0.95. Compared to ruminant sources, cases attributed to poultry sources were typically among adults (odds ratio (OR) = 1.497, 95% confidence intervals (CIs) = 1.211, 1.852), not among males (OR = 0.834, 95% CIs = 0.712, 0.977), in areas with population density of greater than 500 people/km^2 ^(OR = 1.213, 95% CIs = 1.030, 1.431), reported in the winter (OR = 1.272, 95% CIs = 1.067, 1.517) and had undertaken recent overseas travel (OR = 1.618, 95% CIs = 1.056, 2.481). The poultry assigned strains had a similar epidemiology to the unassigned strains, with the exception of a significantly higher likelihood of reporting overseas travel in unassigned strains.

**Conclusions:**

Rather than estimate relative risks for acquiring infection, our analyses show that individuals acquire *C. jejuni *infection from different sources have different associated risk factors. By enhancing our ability to identify at-risk groups and the times at which these groups are likely to be at risk, this work allows public health messages to be targeted more effectively. The rapidly increasing capacity to conduct genetic typing of pathogens makes such traced epidemiological analysis more accessible and has the potential to substantially enhance epidemiological risk factor studies.

## Background

Epidemiological risk factor analyses are used to identify factors that influence the risk of individuals acquiring a particular infection. Such risk factor analyses commonly assume that the risk factors associated with different sources of exposure to infection are homogeneous [1-3]. However, in many cases there are multiple sources of infection and different risk factors may be associated with the different sources. Backward-tracing data on the sources of infection could be used to ascribe different risks to different sources of exposure.

Infection with *C. jejuni *can be acquired from consumption of contaminated food as well as through direct and indirect contact with animal faeces and has multiple hosts including poultry, ruminants and wild birds [4,5]. Recent developments in the typing of *Campylobacter *bacteria permits the tracing of sources of infection for human cases of Campylobacteriosis [6]. *Campylobacter *can be classified by their allelic profile using Multi-Locus-Sequence-Type (MLST) typing techniques [7], which places isolates into specific Sequence Type (ST) profiles. Using STRUCTURE software [8] it is possible to calculate a probability of the ST originating from a particular species [6,9].

Previous studies have identified an association between human *C. jejuni *infection in Scotland and lower social deprivation score (indicating lower social deprivation) and being a child living in an area of lower population density [10]. A recent study in New Zealand [11] typed *C. jejuni *isolates using MLST and used the Asymmetric Island probabilistic genetic attribution model [12] to divide these types into ruminant and poultry origin types. Logistic regression analysis of the two types demonstrated that cases of ruminant origin were more likely to occur in rural areas relative to those of poultry origin [11]. A similar methodology will be used in this paper to build on the risk factor analysis of Bessell et al. [10] by differentiating between the risks associated with different sources of infection. For example, one potential explanation for the association found by Bessell et al. [10] with lower deprivation could be differences in access to outdoor leisure activities. If this were the case, it might result in the less deprived being more exposed to ruminant strains should there be greater exposure to ruminant types in the environment.

By comparing the risk factors that are associated with infection by ruminant or poultry associated types, this paper will investigate the value of genetic data, in this instance MLST, to quantify differences in risks associated with different sources. The following hypotheses will be tested:

1. Infection with ruminant strains is more common in rural areas with a large ruminant population.

2. Infection with ruminant types is more associated with lower deprivation than infection with poultry types.

3. Infection with ruminant types is more common in summer relative to poultry types.

4. Infection with ruminant types is more common among children rather than adults relative to poultry types.

5. Infection with ruminant types is associated with domestic exposures whilst poultry attributed infections more commonly result from exposure to exotic types overseas.

## Methods

### Data

Anonymised reports of laboratory confirmed, passively reported *C. jejuni *infections were collected by staff at Health Protection Scotland (HPS) from the Public Health Teams at the 12 mainland NHS Health Boards that existed in Scotland prior to 2006. Ethical approval for the collection and use of the data was obtained from the Multi-Centre Research Ethics Committee (MREC) in Scotland; additionally, approval for the research was obtained from the Research and Development Committee in each of the NHS Health Boards. Cases that were confirmed between June 2005 and September 2006 were typed using MLST [6,7]. Typing data was linked to epidemiological and demographic data, where available. The data included the postcode sector of the main residence of the case and either the date of onset or more commonly the date of laboratory report. Cases that were part of an outbreak were excluded and of the remainder, 101 cases were missing a verifiable postcode; these were excluded, leaving 3,834 cases. A further 2 cases had no data on gender and 9 had no record of age; these were also removed leaving 3,823 cases.

In a recent study, we collected samples of *C. jejuni *from food and environmental sources including chicken, pate and liver, farms with ruminant livestock, livestock faeces, wild bird faeces and urban areas where animal faeces and humans coincide, such as parks [9]. *C. jejuni *were isolated from these samples and typed using MLST. Subsequently each isolated ST was assigned a probability of originating from a particular source - either poultry, cattle, sheep, wild birds, water and environmental based on their occurrence in each source [6]. The probabilities were assigned using the STRUCTURE software [8]. Each of 441 STs isolated from the 3,451 human cases of *C. jejuni *(372 cases that were infected with *C. coli *were removed from the analysis) was assigned a probability that the ST originated from poultry, cattle, sheep, wild bird and environmental sources as described in Sheppard et al. [6]. STs were assigned to ruminant (cattle and sheep), poultry or wild bird whenever the probability for that species was greater than 0.95; otherwise the case remained unassigned. Very few cases were assigned to environmental or swine origin, so these sources were excluded [6]. Cattle and sheep were merged to form a single ruminant category because Ogden et al. [13] demonstrated that there are no significant differences between probabilities assigned to cattle compared to probabilities assigned to sheep and therefore the two sources are indistinguishable in terms of their *C. jejuni *sequence types.

### Logistic regression

Three separate case-case logistic regression analyses were carried out for all combinations of source of infection assignments. As this is a case-case analysis the group used for the base of comparison in the logistic regression are referred to as 'controls' despite them being incidences of disease:

1. Individuals infected with a poultry assigned type (cases) versus individuals infected with a ruminant assigned type (controls).

2. Individuals infected with an unassigned type (cases) versus individuals infected with a ruminant assigned type (controls).

3. Individuals infected with a poultry assigned type (cases) versus individuals infected with an unassigned type (controls).

As the data points are individual cases, case-specific data could be included. Such data include the age, gender and time of year of laboratory reports. The following putative risk factors were included in these analyses:

• The Carstairs deprivation score of the postcode sector [14] (larger values represent greater deprivation) taken from the 2001 Scottish census [15].

• Easting and northing of the postcode sector centroid.

• Population density (people/km^2^) of the postcode sector using population data from the 2001 Scottish census [15]. This was split to a binary predictor based around a cut-off of 500 people/km^2^.

• Density of cattle, sheep and poultry (head/km^2^) in the postcode sector from the June 2004 agricultural census (EDINA, http://edina.ac.uk/agcensus, 2004 estimates).

• Gender (Female reference level)

• Age: Adult/Child (Adult reference level). Children defined as being 18 and under.

• Season in which infection reported: Summer/Winter (Summer reference level). Summer 15 April to 15 October.

• Reporting of recent overseas travel.

To allow for the clustering of certain predictors at the level of 749 postcode sectors, the postcode sector is entered as a random effect. Furthermore, the data were gathered by the 12 mainland NHS Health Boards, so this was entered as a second random effect. Following univariate screening all predictors that were significant at p < 0.25 were entered into a multivariable model which was subsequently reduced by excluding the least significant predictors in turn until only those which were significant at p < 0.05 remained. The effect of removing predictors on the remaining p-values was monitored. Sensitivity analysis checked for the effect of the source assignment cut -off probability by repeating the analysis for a range of cut-off probabilities from 0.5 to 1 and testing for significant change in the risk factors in the final reduced model. Multilevel logistic regression analysis was carried out using the lme4 package [16] in the R statistical environment [17].

## Results

### Attribution

**Figure 1:**
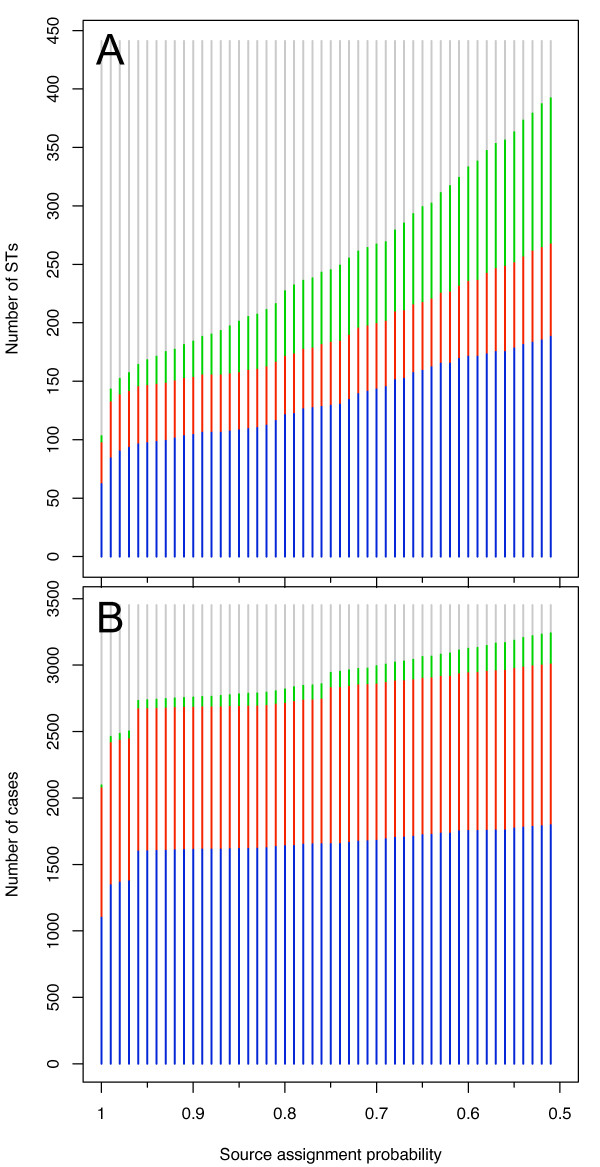
**Source assignment by cut-off probability**. The breakdown of the number of unique ST isolates assigned to each source (Figure 1A) and the numbers of cases assigned to each source (Figure 1B). Poultry sources are represented by blue bars, ruminant sources by red bars, wild bird sources by green bars) and unassigned by grey bars. As the cut-off source probability decreases, the number of unassigned STs and cases declines.

For a cut-off probability close to 1 the majority of STs can not be assigned to a source of origin (Figure 1A). Relative to the increase in number of wild bird STs as the cut-off probability falls, the number of ruminant and poultry assigned STs increases more slowly (Figure 1A). However, the number of cases assigned to each origin is relatively robust to the choice of cut-off probability (Figure 1B). The majority of cases were assigned to poultry sources (46.4%); 31.0% were assigned a ruminant source whilst 20.7% did not have a source with greater than 0.95 probability (Table 1; Figure 1B). Cases resulting from STs attributed to wild bird sources are very few (1.9%). This does not resemble the distribution of STs, the majority of which (61.3%) were not assigned to a source. Overall, there were 2.6 cases per ST, whereas for poultry and ruminant attributed STs there was a mean of 16.5 and 21.8 cases per ST (Table 1).

**Table 1:** The numbers of cases and STs assigned to different sources based upon a probability of greater than 0.95

Attribution	Number of cases (%)	Number of STs (%)	Cases per ST
Poultry	1599 (46.3)	97 (22.0)	16.5

Ruminant	1070 (31.0)	49 (11.1)	21.8

Wild bird	67 (1.9)	22 (5.0)	3.0

Unassigned	715 (20.7)	273 (61.9)	2.6

### Logistic regression analysis

**Figure 2:**
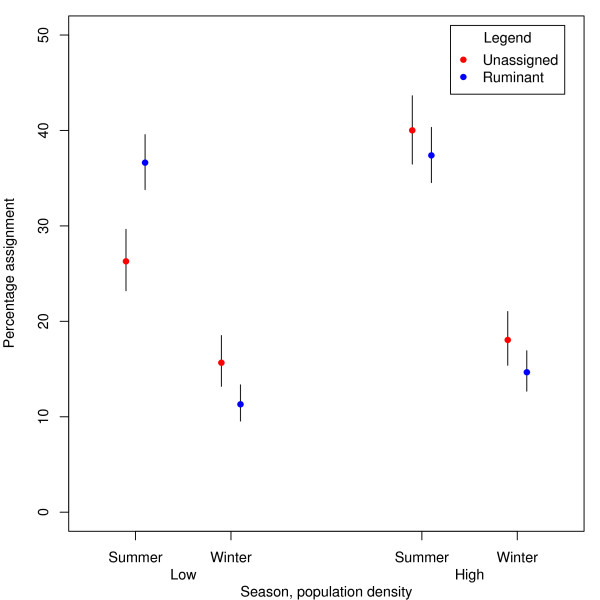
**Case origin by season**. The proportion of cases with unassigned or ruminant origins broken down by season and population density corresponding with the interaction in Table 3. Lines represent 95% binomial confidence intervals.

Compared to ruminant assigned cases, poultry assigned cases are more common in winter, in adults, in females, in postcode sectors with population densities greater than 500 people/km^2 ^and more common in individuals reporting overseas travel (Table 2). Relative to ruminant assigned cases, unassigned cases were also more common in winter and in adults, more common in individuals reporting overseas travel (Table 3), and there was an interaction between season and population density such that there was a stronger seasonal effect among unassigned cases in areas of high population density (Table 3; Figure 2). The only significant risk factor for being a poultry assigned case compared to an unassigned case was overseas travel (OR = 0.318, 95% CIs = 0.231, 0.439) and therefore no further results are presented. Additionally the results were tested for sensitivity to the choice of cut off probability for assignment of STs to a source, but adjusting this had no significant effect on the model.

**Table 2:** Logistic regression comparing risk factors for being infected by a ruminant attributed type (control) with those for a poultry attributed type (case)

Predictor	Unit	OR (95% CIs)	z-value	p-value
Intercept		NA	0.213	0.831

Age	Child	1	-	-
	
	Adult	1.497 (1.211, 1.852)	3.772	< 0.001

Season	Summer	1	-	-
	
	Winter	1.272 (1.067, 1.517)	2.678	0.007

Sex	Female	1	-	-
	
	Male	0.834 (0.712, 0.977)	-2.248	0.025

Overseas travel	No	1		
	
	Yes	1.618 (1.056, 2.481)	2.212	0.021

Population dens	< = 500/km^2^	1	-	-
	
	> 500/km^2^	1.213 (1.030, 1.431)	2.313	0.027

**Table 3:** Logistic regression comparing risk factors for being infected by a ruminant attributed type (control) with those for an unassigned type (case).

Predictor	Unit	OR (95% CIs)	z-value	p-value
Intercept		NA	8.094	< 0.001

Age	Child	1	-	-
	
	Adult	1.524 (1.156, 2.008)	2.994	0.003

Season	Summer	1	-	-
	
	Winter	1.919 (1.399, 2.632)	4.035	< 0.001

Overseas travel	No	1		
	
	Yes	4.808 (3.165, 7.299)	7.377	< 0.001

Population dens	< = 500/km^2^	1	-	-
	
	> 500/km^2^	1.359 (1.071, 1.724)	2.520	0.012

Season * pop.dens	Summer * < = 500/km^2^	1	-	-
	
	winter * > 500/km^2^	0.605 (0.395, 0.926)	-2.316	0.021

## Discussion

By using the MLST technique to attribute isolates from *C. jejuni *cases to host sources [6], this paper has demonstrated that risk factors for infection depend upon the source of the pathogen. Whilst there is a range of potential sources of *C. jejuni *infections, this paper has demonstrated that human infections of *C. jejuni *that are attributable to sources in ruminants are more seasonal and occur more in rural areas than those assigned to poultry sources. Those that were unassigned had very similar epidemiologies to the poultry attributable types.

The work of Sheppard et al. [6] on assigning source probabilities to individual STs has made this analysis possible and it demonstrates that the majority of human cases were attributable to sources in poultry and ruminants or were unassigned (Table 1). However, the majority of STs were not assigned to a source of infection with a probability of greater than 95%. This is in part reflects the large number of STs that represented a small proportion of human infections (Table 1 Figure 1), and suggests that there are either a large number of *C. jejuni *to which humans have low susceptibility or to which humans are rarely exposed. Consequently, changes in human behaviour or environmental exposures could result in exposure to a large additional pool of bacteria. Twenty-two *C. jejuni *STs were assigned to wild bird origins, but there were only in 67 reported human cases assigned to an origin in wild birds. This suggests that whilst wild birds are a reservoir there is little mechanism for human exposure, although exposure to preschool children in playgrounds has been suggested elsewhere [18].

The comparisons of poultry attributed cases, ruminant attributed cases and unassigned cases (Tables 2 and 3) showed that ruminant assigned types were more common in children in rural areas in summertime. This may reflect a tendency to play outdoors in the summertime coupled with poor hygiene after playing outdoors. Strachan et al. [19] find similar results and attribute the differences to the consumption of contaminated chicken in urban areas and playing outdoors in rural areas. These findings are similar to those from New Zealand [11], although our larger sample size has enabled us to show that younger age groups in rural areas are at greater risk of infection with a ruminant types in addition to the effect of season. Thus, the heterogeneities in exposure to infection of *C. jejuni *are consistent across different countries, with similar mechanisms of infection occurring in all, despite the fact that the most common ST in New Zealand that is associated with poultry (ST474) differs from that in Scotland (ST257).

Previous studies [1,10] have identified an association with increased incidence in younger individuals that live in more rural settings. This paper suggests that this is likely to be the result of infection with ruminant types, thus underlining the importance of identifying different sources of infections. Here, the density of the human population rather than the density of cattle and sheep has been identified as the measure of risk for infection with ruminant strains. This suggests that either population density is a better measure of exposure to ruminant sources or that it is some property of rural areas that determines the risk. One such property has been demonstrated to be consumption of water from untreated sources [20]. It is likely that consumption of water from private water sources will be greater in rural areas with lower population densities. ST45 was identified as a type that was associated with surface water sources during a period in the summer [20], however, in this study ST45 was attributed to sources in poultry.

This study did not demonstrate any difference in the risk associated with deprivation for different sources of infection. The relationship between campylobacteriosis and deprivation has been noted in Scotland [10], Denmark [1] and New Zealand [21], but the non-significance of deprivation in this study suggests that deprivation does not influence exposure to environmental sources.

The unassigned types had similar epidemiologies to the poultry types with the consequence that the only significant risk factors for being infected with a poultry rather than an unassigned type was overseas travel. This suggests that the majority of these unassigned types had a similar epidemiology to the poultry types, but insufficient isolates were found in the source assignment to demonstrate their origin and the association with overseas travel suggests that these may be exotic types. Bessell et al. [10] describe a higher likelihood of reporting infection in areas of lower deprivation and lower population density. These analyses show that the effect of rurality may be the signature of the ruminant origin cases.

By using a case-case approach this study did not seek to estimate population level risk of exposure. Rather this study analysed the subgroup of the population that has already been infected, with the principal risk factor being social deprivation [10]. Case-case analysis is a means of comparing risk factors within this sub-group of the population that has acquired infection [22] and has been employed elsewhere for comparing risk factors for infection between sources of *C. jejuni *[23]. As such, social deprivation remains the principal population level determinant of infection with *C. jejuni *but these analyses demonstrate that this does not vary between sources of infection.

## Conclusions

Our results have demonstrated that over and above the previously demonstrated risk factors for infection at the population level [10], there are different risk factors for infection depending upon the sources of exposure to infection. Therefore, it is important to account for the source of infection in public health planning. The individuals that report infection depend upon the source of *C. jejuni*, with ruminant exposures more common among the young males in rural areas. For common genetic types, this analysis could be expanded to examine transmission routes that are specific to individual strains. By enhancing our ability to identify at-risk groups and the likely times at which these groups are at risk, public health messages can be targeted more effectively. The rapidly increasing capacity to conduct genetic typing of pathogens makes such traced epidemiological analysis more accessible and has the potential to substantially enhance epidemiological risk factor studies.

## Competing interests

The authors declare that they have no competing interests.

## Authors' contributions

PRB carried out data analysis and drafted the manuscript. OR, NJCS and KJF participated in the cleaning and processing of the MLST data. GTI assisted with statistical analysis and participated in the drafting of the manuscript. ASP and JMC gathered, participated in the cleaning and processing of the *Campylobacter *case data and assisted with specific public health aspects of the study. SWJR participated in the design and coordination of the study. LM participated in the design and guidance of the study and drafting of the manuscript. All authors read and approved the final manuscript.

## Pre-publication history

The pre-publication history for this paper can be accessed here:

http://www.biomedcentral.com/1471-2334/12/80/prepub

## References

[B1] Ethelberg S Simonsen J Gerner-Smidt P Olsen KE Molbak K Spatial distribution and registry-based case-control analysis of Campylobacter infections in Denmark, 1991-2001 Am J Epidemiol 2005 162 10 1008 1015 10.1093/aje/kwi316 16207804

[B2] Halliday JE Chase-Topping ME Pearce MC McKendrick IJ Allison L Fenlon D Low C Mellor DJ Gunn GJ Woolhouse ME Herd-level risk factors associated with the presence of Phage type 21/28 E. coli O157 on Scottish cattle farms BMC Microbiol 2006 6 99 10.1186/1471-2180-6-99 17140453 PMC1713242

[B3] Bessell PR Shaw DJ Savill NJ Woolhouse ME Statistical modeling of holding level susceptibility to infection during the 2001 foot and mouth disease epidemic in Great Britain Int J Infect Dis 2010 14 3 e210 e215 10.1016/j.ijid.2009.05.003 19647465

[B4] Horrocks SM Anderson RC Nisbet DJ Ricke SC Incidence and ecology of Campylobacter jejuni and coli in animals Anaerobe 2009 15 1-2 18 25 10.1016/j.anaerobe.2008.09.001 18849005

[B5] Mullner P Spencer SE Wilson DJ Jones G Noble AD Midwinter AC Collins-Emerson JM Carter P Hathaway S French NP Assigning the source of human campylobacteriosis in New Zealand: a comparative genetic and epidemiological approach Infect Genet Evol 2009 9 6 1311 1319 10.1016/j.meegid.2009.09.003 19778636

[B6] Sheppard SK Dallas JF Strachan NJ MacRae M McCarthy ND Wilson DJ Gormley FJ Falush D Ogden ID Maiden MC Campylobacter genotyping to determine the source of human infection Clin Infect Dis 2009 48 8 1072 1078 10.1086/597402 19275496 PMC3988352

[B7] Maiden MC Bygraves JA Feil E Morelli G Russell JE Urwin R Zhang Q Zhou J Zurth K Caugant DA Multilocus sequence typing: a portable approach to the identification of clones within populations of pathogenic microorganisms Proc Natl Acad Sci USA 1998 95 6 3140 3145 10.1073/pnas.95.6.3140 9501229 PMC19708

[B8] Pritchard JK Stephens M Donnelly P Inference of population structure using multilocus genotype data Genetics 2000 155 2 945 959 10835412 10.1093/genetics/155.2.945 PMC1461096

[B9] Sheppard SK Dallas JF Macrae M McCarthy ND Sproston EL Gormley FJ Strachan NJ Ogden ID Maiden MC Forbes KJ Campylobacter genotypes from food animals, environmental sources and clinical disease in Scotland 2005/6 Int J Food Microbiol 2009 134 1-2 96 103 10.1016/j.ijfoodmicro.2009.02.010 19269051 PMC3985063

[B10] Bessell PR Matthews L Smith-Palmer A Rotariu O Strachan NJ Forbes KJ Cowden JM Reid SW Innocent GT Geographic determinants of reported human Campylobacter infections in Scotland BMC Public Health 2010 10 423 10.1186/1471-2458-10-423 20633277 PMC2918555

[B11] Mullner P Shadbolt T Collins-Emerson JM Midwinter AC Spencer SE Marshall J Carter PE Campbell DM Wilson DJ Hathaway S Molecular and spatial epidemiology of human campylobacteriosis: source association and genotype-related risk factors Epidemiol Infect 2010 138 10 1372 1383 10.1017/S0950268809991579 20141645

[B12] Wilson DJ Gabriel E Leatherbarrow AJ Cheesbrough J Gee S Bolton E Fox A Fearnhead P Hart CA Diggle PJ Tracing the source of campylobacteriosis PLoS Genet 2008 4 9 e1000203 10.1371/journal.pgen.1000203 18818764 PMC2538567

[B13] Ogden ID Dallas JF MacRae M Rotariu O Reay KW Leitch M Thomson AP Sheppard SK Maiden M Forbes KJ Campylobacter excreted into the environment by animal sources: prevalence, concentration shed, and host association Foodborne Pathog Dis 2009 6 10 1161 1170 10.1089/fpd.2009.0327 19839759 PMC3985071

[B14] Carstairs V Morris R Deprivation and health in Scotland Health Bull (Edinb) 1990 48 4 162 175 2394583

[B15] UKBorders Service http://www.edina.ac.uk/

[B16] Bates D Maechler M Bin D lme4: Linear mixed-effects models using S4 classes 2011

[B17] R Development Core Team R: A language and environment for statistical computing 2008 Vienna, Austria: R Foundation for Statistical Computing

[B18] French NP Midwinter A Holland B Collins-Emerson J Pattison R Colles F Carter P Molecular epidemiology of Campylobacter jejuni isolates from wild-bird fecal material in children's playgrounds Appl Environ Microbiol 2009 75 3 779 783 10.1128/AEM.01979-08 19047378 PMC2632120

[B19] Strachan NJ Gormley FJ Rotariu O Ogden ID Miller G Dunn GM Sheppard SK Dallas JF Reid TM Howie H Attribution of campylobacter infections in northeast Scotland to specific sources by use of multilocus sequence typing J Infect Dis 2009 199 8 1205 1208 10.1086/597417 19265482 PMC3985119

[B20] Sopwith W Birtles A Matthews M Fox A Gee S Painter M Regan M Syed Q Bolton E Identification of potential environmentally adapted Campylobacter jejuni strain, United Kingdom Emerg Infect Dis 2008 14 11 1769 1773 10.3201/eid1411.071678 18976567 PMC2630731

[B21] Rind E Pearce J The spatial distribution of campylobacteriosis in New Zealand, 1997-2005 Epidemiol Infect 2010 138 10 1359 1371 10.1017/S095026881000018X 20141648

[B22] McCarthy N Giesecke J Case-case comparisons to study causation of common infectious diseases Int J Epidemiol 1999 28 4 764 768 10.1093/ije/28.4.764 10480708

[B23] Gillespie IA O'Brien SJ Frost JA Adak GK Horby P Swan AV Painter MJ Neal KR A case-case comparison of Campylobacter coli and Campylobacter jejuni infection: a tool for generating hypotheses Emerg Infect Dis 2002 8 9 937 942 12194770 10.3201/eid0809.10.3201/eid0809.010187 PMC2732536

